# Intraspecific variation in Janzen–Connell effect is mediated by stress and plant–soil feedbacks

**DOI:** 10.1002/ece3.11614

**Published:** 2024-06-30

**Authors:** Libing Pan, J. Aaron Hogan, Xiaoyang Song, Wenfu Zhang, Huaze Zhou, Zhonglin Chen, Jie Yang, Min Cao

**Affiliations:** ^1^ CAS Key Laboratory of Tropical Forest Ecology, Xishuangbanna Tropical Botanical Garden Chinese Academy of Sciences Mengla China; ^2^ University of Chinese Academy of Sciences Beijing China; ^3^ Department of Biology University of Florida Gainesville Florida USA; ^4^ Mengla Institute of Conservation Xishuangbanna Administration of Nature Reserves Mengla China

**Keywords:** biomass, competition, drought, experiment, intraspecific, plant–soil feedback, Xishuangbanna

## Abstract

Janzen–Connell (JC) effects, hypothesized to be partially driven by negative plant–soil feedbacks (PSFs), are considered to be a key mechanism that regulates tropical forest plant diversity and coexistence. However, intraspecific variation in JC effects may weaken this mechanism, with the strength of PSFs being a potentially key variable process. We conducted a manipulated experiment with seedlings from two populations of *Pometia pinnata* (Sapindaceae), a tropical tree species in southwest China. We aimed to measure the intraspecific difference in PSF magnitude caused by inoculating the soil from different *P. pinnata* source populations and growing seedlings under differing light intensity and water availability treatments, and at varying plant densities. We found negative PSFs for both populations with the inoculum soil originating from the same sites, but PSFs differed significantly with the inoculum soil from different sites. PSF strength responded differently to biotic and abiotic drivers; PSF strength was weaker in low moisture and high light treatments than in high moisture and low light treatments. Our study documents intraspecific variation in JC effects: specifically, *P. pinnata* have less defenses to their natively‐sourced soil, but are more defensive to the soil feedbacks from soil sourced from other populations. Our results imply that drought and light intensity tended to weaken JC effects, which may result in loss of species diversity with climate change.

## Graphical abstract

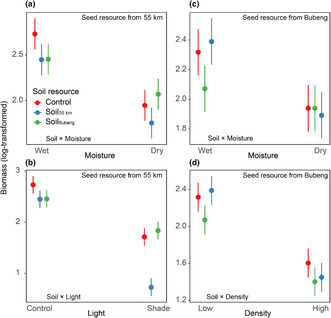

## 1 INTRODUCTION

Understanding how ecological processes vary with drivers of climate change (e.g., increasing temperature and drought) is critical to predicting future biodiversity responses to environmental change (Liu & He, 2021). Janzen–Connell (JC) effects (oftentimes referred to under the broader term, conspecific negative density dependence) are one of the most important processes which permit the stable coexistence of plant diversity in highly diverse ecosystem. The JC theory posits that specialized enemies–either herbivores or soil microorganisms–reduce the fitness of individuals growing at higher conspecific densities and closer to conspecific adults (Connell, 1971; Janzen, 1970). Specialist herbivores can only partly explain JC effects, making the need to further elucidate the role of soil microorganisms (i.e., bacteria and fungi) and plant–soil feedbacks (PSFs) in JC interactions imperative. PSFs are defined as reciprocal interactions between plants and soils, oftentimes mediated by soil biota, which are important agents of JC effect (Eppinga et al., 2018; Semchenko et al., 2022).

Previous studies about PSFs have mostly focused on species‐level interactions (Kulmatiski et al., 2008); few studies have focused on the factors affecting PSF variability within a species (but see Liu et al., 2015). Genetic differences could, in theory, decrease the strength of PSFs and thereby weaken JC effects (Liu et al., 2015), because some soil‐borne pathogens have coevolved with plant populations (i.e., genotypes), potentially limiting some soil biota to certain individuals of a species (Liu & He, 2019). Thus, it is important to know how the variation of PSFs within species may be promoting species coexistence through intraspecific variation in JC effects strength (Shao et al., 2018).

Forest ecosystem is undergoing rapid climatic changes which include increasing frequency and severity droughts in many regions (Brodribb et al., 2020; McDowell et al., 2020). Drought not only causes physiological plant stress, but also modulates biotic interaction which can further suppress plant performance (Comita & Engelbrecht, 2009; Fichtner et al., 2020; O'Brien et al., 2017; Song et al., 2021; Uriarte et al., 2010). For example, in a tropical forest in Panama, Janzen–Connell negative density dependent effects were shown to be stronger in wetter than drier years, thereby promoting diversity at high soil moisture but promoting species dominance at low soil moisture (Lebrija‐Trejos et al., 2023); yet the mechanism behind this pattern is unclear. For example, drought can create unsuitable conditions for soil microorganism populations and potentially weaken the infectiousness and pathogenicity of soil‐borne plant pathogens (Liu & He, 2019; Milici et al., 2020), thereby decreasing the PSFs which may result in Janzen–Connell effects. On the other hand, the low soil moisture levels under drought may decreasing host plant defenses making them more susceptible to soil‐residing microorganisms, thereby strengthening the PSFs which result in Janzen–Connell processes (Jactel et al., 2012; Liu & He, 2021). Thus, the strength of PSFs in relation to environmental drivers can help understand variation in JC effects and their ecological consequences.

Light conditions may also modulate plant‐microbe and plant–plant interaction strength, thereby affecting PSFs. High light conditions can reduce the activity of soil‐borne plant pathogens (Augspurger & Kelly, 1984), but might also increase plant photosynthesis and allocation to defenses (Ballaré, 2014). On the contrary, relatively low light not only affect plant growth by directly decreasing plant photosynthesis, but may also suppress pathogen resistance (McCarthy‐Neumann & Kobe, 2008). Thus, negative PSFs may be weakened by high light conditions, and strengthened under low light conditions (McCarthy‐Neumann & Ibanez, 2013).

Soil feedbacks occur simultaneously and concurrently with intraspecific competition–especially for seedlings–as dispersal limitation results in the highest seedling density near parent trees (Shao et al., 2018; Terborgh, 2020). A meta‐analysis which quantified the effects of interspecific competition and PSF suggested that competition increases negative PSF strength (Lekberg et al., 2018). Similarly, intraspecific competition may reduce resource acquisition of seedlings, making them more‐easily infected by soil pathogens than seedlings with lower levels of intraspecific competition (Augspurger & Kelly, 1984). Moreover, interactions between soil‐borne pathogens and tropical seedling intraspecific competition have been shown to be species‐specific and variable (Gripenberg et al., 2014). However, conversely a recent meta‐analysis claimed that the interaction between PSFs and competition is overall non‐significant (Beals et al., 2020). While the strength of PSFs under varying competition scenarios may be context dependent—for example, less competition for water may occur when soil moisture is sufficient, but declining water supply can cause strong conspecific competition for water (Lamont et al., 1993; Meinzer et al., 1999; O'Brien et al., 2017). Moreover, PSF strength may not only directly increase physiological plant stress, but also may increase indirect plant stress due to increased sensitivity to natural enemies.

We conducted a manipulated PSF experiment with seedlings of a tropical tree species *Pometia pinnata* J.R.Forst & G.Forst. (Sapindaceae) in southwest China to test the interaction between environmental drivers (soil moisture and light) and PSF strength resulting from JC effects by manipulating light intensity, water availability, and seedling density. Using a reciprocal transplant methodology with seedlings and soil sourced from two different populations, we asked: (1) Does *P. pinnata* experience PSFs resulting from JC effects? If so, does PSF strength differ in response to soil from different plant population? and (2) How do competition (i.e., seedling density), light and water conditions affect PSF strength? We expected that (1) soil biota from the same population should show strong JC effects, while the soil biota from different population would cause similar or weaker JC effects, and that (2) low light condition should increase JC effects, but competition and low soil moisture likely decrease JC effects, and that variation should be soil source dependent.

## 2 METHODS

### 2.1 Study site and study species

The experiment was conducted in the Xishuangbanna, Yunnan Province, China. Xishuangbanna has a tropical monsoon climate, with a pronounced wet season between May and October and a dry season between November and April (Cao et al., 2006). The average annual temperature is 21°C, with an average annual rainfall of approximately 1500 mm, approximately 80% of the annual rainfall occurs in the rainy season.

*Pometia pinnata* J.R.Forst & G.Forst. (Sapindaceae) is one of the dominant late‐successional tree species in the tropical seasonal rainforest in this area (Wu et al., 1987). The fruit supports many animal species, and is one of the most important fundamental tree species of tropical rainforest (Yan & Cao, 2008).

#### 2.1.1 Seed collection and germination

We collected seeds from two population of *P. pinnata* that located in Bubeng (21°37′08″ N, 101°35′07″ E) and “55 km” (21°57′40″ N, 101°12′01″ E), located within the Xishuangbanna National Nature Reserve in September of 2019. Seeds were collected from three or more mature individuals growing in each forest and transported to the greenhouse in Xishuangbanna Tropical Botanical Garden (XTBG; 21°55′30″ N, 101°15′59″ E). Seed coats were removed by hand; seed surfaces were soaked for 15 min in a 1% potassium permanganate solution to sterilize them, then seeds were germinated in moistened sterilized sand. Most seeds germinated within 2 weeks. Then, germinated seedlings with similar sizes were transplanted in October 2019.

#### 2.1.2 Soil collection

Soil was collected from three areas for the experiment. We collected the soil under *P. pinnata* adult trees in Bubeng and 55 km, respectively, for each site, we selected three adult trees (DBH > 40 cm), we removed all the litters and collected the surface soil (0–5 cm) within a 2‐m radius from the tree. We also collected the immature soil at >2 m depth in the rubber plantation in the XTBG.

#### 2.1.3 Experimental design

The experiment was conducted in the greenhouse in XTBG. In total 240 pots (24 cm diameter × 27 cm height) were prepared; pots were filled with 9 L of immature rubber plantation deep soil. Before the start of the experiment, all pots were fully watered, and 500 mL Hoagland nutrient solution (calcium nitrate 945 mg/L, potassium nitrate 607 mg/L, ammonium phosphate 115 mg/L, magnesium sulphate 493 mg/L, iron salt solution 2.5 mL/L, trace elements 5 mL/L, pH 6.0) was added to supply the same soil nutrient level for each pot.

**FIGURE 1:**
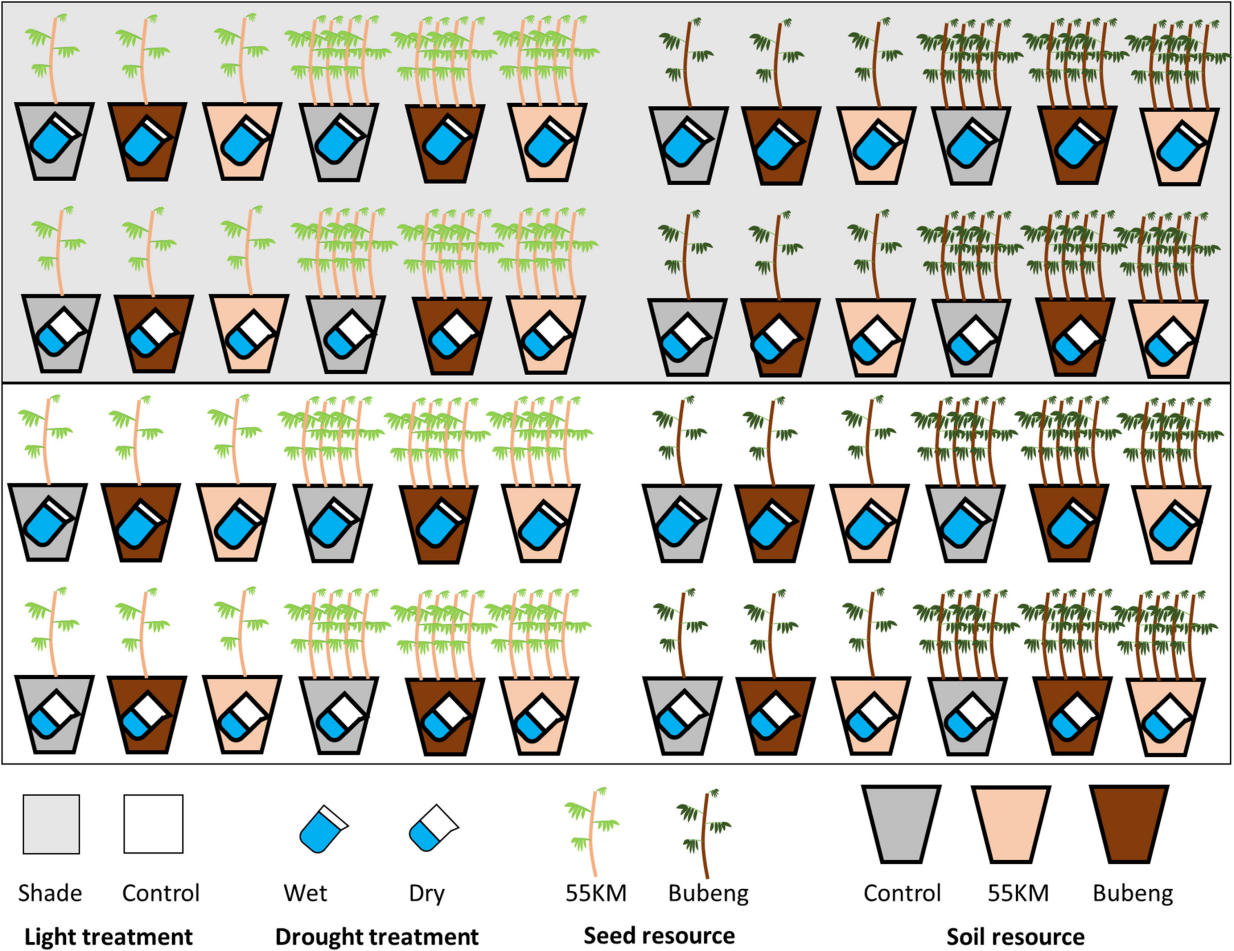
Illustration of the experimental setup. Gray and white background colors indicted shade and control light treatments. The seedlings with different colors indicated different populations from either 55 km or Bubeng. Different quantity of water indicated control and drought treatments. Pots with different colors indicated different soil recourses: gray pot represents soil from common garden, orange pot represents soil from 55 km, and brown pot represents soil from Bubeng.

Beginning in October 2019, seedlings were transplanted in a fully randomized experimental design to test whether conspecific competition affect conspecific negative density dependence (i.e., JC effects) and how light and moisture conditions modulate these effects (Figure 1).

We planted a total of 384 seedlings (192 from each population) into 240 pots. For each population, we included planting density, soil, moisture and light as experimental treatments. For each density × light × soil × moisture treatment, there were 8 seedling replicates. For the density treatment, we planted one seedling per pot as the low‐density treatment, and four seedlings per pot as the high‐density treatment. For soil treatment, we added 50 g of soil from either one of the three different soil sources (soil under the *P. pinnata* adult tree in Bubeng and 55 km, and immature soil in the garden) to the top of the experimental pot. For soil moisture treatments, we included wet and dry soil conditions, based on the past 10 years of rainfall in Xishuangbanna. For the wet treatment, we irrigated 2400 mL week^−1^ to simulate rainfall conditions during in the rainy season (approx. 200 mm month^−1^). For the dry treatment we irrigated 600 mL week^−1^ (a 75% reduction from the wet treatment) to simulate rainfall conditions during the regular dry season (approx. 50 mm month^−1^). For the light treatment, we used black shading net to set two levels light condition, ~20% full light as the control to simulate light conditions in a forest gap and ~4% as shade treatment to simulate typical light conditions in the forest understory. Seedlings that died within 1 week after transplanting were replaced with new seedlings of the same site.

At 24 weeks from transplanting, all seedlings were harvested and measured for seedling biomass. Two seedlings from 55 km population died during the experiment, so we harvested 190 seedlings from 55 km population and 192 seedlings from Bubeng population.

### 2.2 Statistical analyses

We defined seedling growth as the total plant biomass at the time of harvest. First, we use linear models to model the seedling growth rates (biomass gain per unit time) for each of the two seedling populations. First, we fit the full model with all explanatory variables including density, moisture, soil and light treatments, including all possible two‐, three‐ and four‐ way interactions. We set immature soil, high light intensity, wet soil moisture, and low seedling density as reference level for each treatment. Seedlings were inoculated by the soil from same site (for example, seedlings from 55 km and inoculum soil from 55 km) and different site (for example, seedling from 55 km but inoculum soil from Bubeng), respectively. Then we used stepwise model selection to choose the best‐fitting model based on AIC (Burnham & Anderson, 2002). All the data analyses were conducted in the R environment for statistical computing v 4.1.3 (R Core Team, 2022).

## 3 RESULTS

Overall, we found both 55 km and Bubeng seedlings showed similar responses to site inoculum soil treatments, seedling density, moisture and light. That is, both seedling populations showed lower biomass when treated with their native‐site inoculum soil, competition, drought and shade treatment (Tables 1 and 2). However, the two populations had different responses to the different site soil inocula. The soil from Bubeng had a significantly negative effect on the biomass of 55 km seedlings but the soil from 55 km had similar effect on Bubeng seedlings compared to control treatment.

**TABLE 1:** The results of best model testing the effects of soil resource (control, soil_55 km_: 55 _km_‐site inoculum soil treatment and soil_Bubeng_: Bubeng‐site inoculum soil treatment), soil moisture (wet vs. dry), light treatment (control vs. shade), density treatment (low vs. high), and their interactions on biomass production of seedlings from 55 _km_ population.

Predictors	Biomass (log‐transformed)		
	Estimates	CI	*p*
Intercept	2.73	2.56 to 2.90	**<.001**
Soil_55 km_	−0.28	−0.49 to −0.07	**.009**
Soil_Bubeng_	−0.28	−0.49 to −0.07	**.010**
Density_high_	−0.79	−0.97 to −0.62	**<.001**
Light_shade_	−1.02	−1.26 to −0.78	**<.001**
Moisture_dry_	−0.78	−1.02 to −0.54	**<.001**
Soil_55 km_: Light_shade_	−0.70	−1.01 to −0.40	**<.001**
Soil_Bubeng_: Light_shade_	0.40	0.10 to 0.70	**.009**
Density_high_: Light_shade_	0.51	0.26 to 0.75	**<.001**
Soil_55 km_: Moisture_dry_	0.09	−0.21 to 0.39	.554
Soil_Bubeng_: Moisture_dry_	0.40	0.10 to 0.69	**.009**
Density_high_: Moisture_dry_	0.11	−0.14 to 0.35	.392
Light_shade_: Moisture_dry_	0.62	0.28 to 0.97	**<.001**
Soil_55 km_: Light_shade_: Moisture_dry_	0.02	−0.41 to 0.44	.943
Soil_Bubeng_: Light_shade_: Moisture_dry_	−0.61	−1.03 to −0.19	**.005**
Density_high_: Light_shade_: Moisture_dry_	−0.24	−0.59 to 0.10	.164
Observations	190		
*R* ^2^/*R* ^2^ adjusted	.814/.798		

Bold values indicated *p*‐value < .05.

**TABLE 2:** The results of best model testing the effects of soil resource (control, soil_Bubeng_: Bubeng‐site inoculum soil treatment and soil_55 km_: 55 _km_‐site inoculum soil treatment), soil moisture (wet vs. dry), light treatment (control vs. shade), density treatment (low vs. high), and their interactions on biomass production of seedlings from Bubeng population.

Predictors	Biomass (log‐transformed)		
	Estimates	CI	*p*
Intercept	2.32	2.16 to 2.48	**<.001**
Soil_Bubeng_	−0.25	−0.42 to −0.07	**.007**
Soil_55 km_	0.07	−0.11 to 0.25	.427
Density_high_	−0.71	−0.92 to −0.51	**<.001**
Light_shade_	−1.10	−1.27 to −0.94	**<.001**
Moisture_dry_	−0.38	−0.58 to −0.17	**<.001**
Soil_Bubeng_: Density_high_	0.04	−0.16 to 0.25	.676
Soil_55 km_: Density_high_	−0.23	−0.43 to −0.02	**.032**
Density_high_: Light_shade_	0.67	0.43 to 0.90	**<.001**
Soil_Bubeng_: Moisture_dry_	0.25	0.04 to 0.45	**.019**
Soil_55 km_: Moisture_dry_	−0.12	−0.33 to 0.09	.251
Density_high_: Moisture_dry_	−0.00	−0.24 to 0.24	.989
Light_shade_: Moisture_dry_	0.49	0.26 to 0.73	**<.001**
Density_high_: Light_shade_: Moisture_dry_	−0.31	−0.65 to 0.02	.066
Observations	192		
*R* ^2^/*R* ^2^ adjusted	.716/.695		

Bold values indicated *p*‐value < .05.

**FIGURE 2:**
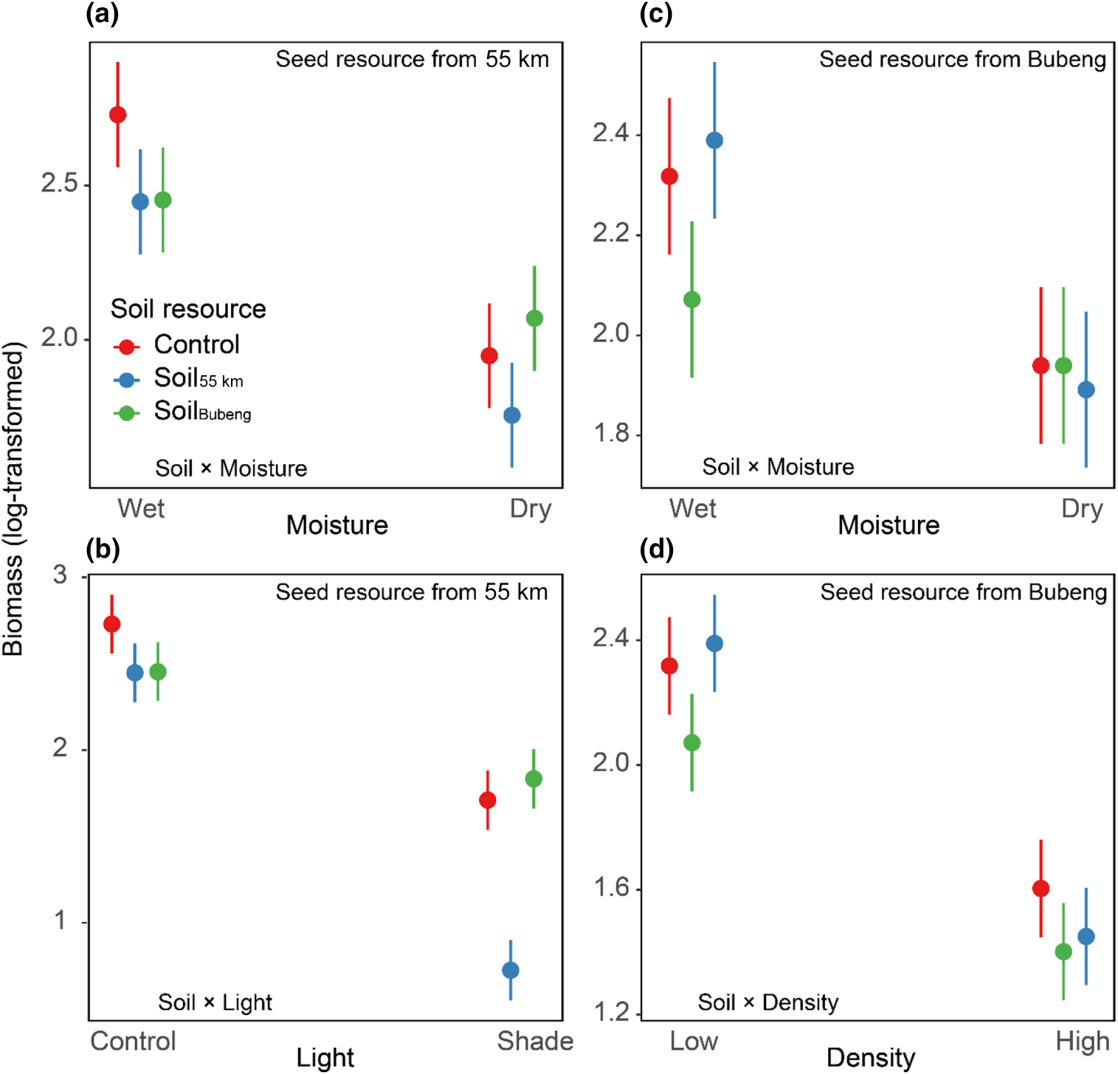
The modeled value of biomass of seedlings in different treatment combination from 55 km and Bubeng populations. The log‐transformed biomass of seedlings in 55 km population (a) in different soil resource and moisture treatment; (b) in different soil resource and light treatment. The log‐transformed biomass of seedlings in Bubeng population (c) in different soil resource and moisture treatment; (d) in different soil resource and density treatment.

Total seedling biomass was significantly affected by the interaction between soil treatment and abiotic and biotic stresses (Figure 2). Specially, the drought treatment decreased the strength of the negative effect of soil from Bubeng on the biomass of 55 km seedlings (Figure 2a). The shade treatment strengthened the negative effect of soil from e55 km on the biomass of 55 km seedlings (Figure 2b), but weakened the negative effect of soil from Bubeng on the biomass of 55 km seedlings. The drought treatment weakened the negative effect of soil from 55 km on the biomass of Bubeng seedlings (Figure 2c). The high seedling density strengthened the negative effect of soil from 55 km on the biomass of Bubeng seedlings (Figure 2d).

## 4 DISCUSSION

JC effects are widely considered to be the key drivers of conspecific negative density dependence (Hülsmann et al., 2021), which are widely thought to promote tropical forest species diversity and coexistence (Lebrija‐Trejos et al., 2023; Wright, 2002). We evaluated the potential for PSFs resulting from intraspecific JC effects by inoculating the soil from different *P. pinnata* plant populations and conducting a seedling growth experiment. We test how PSFs vary with different levels of light intensity, water availability, and seedling density, finding that PSF strength was significantly affected by the inoculum soil from different sites and that PSF strength varies across ranging biotic and abiotic conditions.

The few studies that have examined the intraspecific variation in conspecific interactions suggest stronger negative interactions between more‐closely related individuals (Shao et al., 2018). Liu et al. (2015) confirmed that intraspecific PSFs variation was related to the genetic distance between the target population and the populations where soil was collected. Our results suggest another potential pattern– that genotypes have less defenses to the soil‐borne pathogens in their own specific soil, but the genotypes may have differential defenses to the soil biota in soil collected from other plant populations (Figure 2). This may because soil pathogens are adapted to the subspecies, or genotype level, and different soil pathogens may have different degrees of specificity in how they infect plants and affect their performance (i.e., growth and survival) (Liu & He, 2019). This result suggests that JC effects may act as a mechanism for genetic diversification that could increase the genetic diversity within species, thereby promoting diversity.

Not surprisingly, we found a significant negative effect for soil feedbacks using soil from same population on the growth of *P. pinnata*, which was in line with previous conspecific PSF experiments (Kulmatiski et al., 2008; McCarthy‐Neumann & Kobe, 2010). Moreover, both biotic (intraspecific competition) and abiotic (drought and light) stress not only directly suppressed the growth of *P. pinnata*, which in line with previous studies using other plant species (Lekberg et al., 2018; O'Brien et al., 2017; Xi et al., 2019), but also affected the strength of PSFs (Tables 1 and 2). Specifically, drought weakened, but light limitation strengthened negative PSFs when using inoculum soil from the same site. This result suggests that low soil moisture may suppress the activities of soil pathogens more than weakening *P. pinnata* defense ability. Thus, if JC effects are integral to maintaining diversity, the increasing frequency and severity of drought with climate change may decrease species diversity at community level as drought may cause weaker JC effects (Lebrija‐Trejos et al., 2023). On the other hand, deep shade could maintain high species diversity by strengthening JC effects; however, in many places (including the study region of Xishuangbanna), global climate change is resulting in increases in radiation intensity (Kramer et al., 2021). The increasing frequency of human and natural disturbance could create more tree fall and canopy gaps (Carnicer et al., 2011; Peterson, 2000), which would in turn increase light recourse to the forest understory, which may reduce JC effect and reduce species diversity at community level.

We also found that the interaction between environmental stressors and soil feedbacks was not consistent across soils from different sites (Tables 1 and 2). This result may indicate diverging interactions between soil biota from different site and *P. pinnata* population under climatic changes (Corlett & Westcott, 2013). The different response of *P. pinnata* to the soil from two different sources have potential value in the forest restoration efforts which highlights the importance of population selection in the seeds or seedlings for planted trees. Ex‐situ conservation may also need to consider mixed plantations close to natural forest stands, other than monoculture plantation to reduce JC effect strength within and among different genotypes.

Despite the evidence for environmental stress driving PSF strength and intraspecific variation in JC effects, further studies are still needed. First, deepening our understanding of the mechanisms behind PSFs, such as the composition of the soil pathogen community in different site and under different stress is needed, which could potentially be addressed using DNA sequencing of soils and fungal functional trait data (Põlme et al., 2020). Second, more experimental gradients of stress are needed to detect the potential non‐linear responses of PSFs (Kreyling et al., 2018). Lastly, more PSF experiments that consider interactions with abiotic factors are needed for more species and communities to find general patterns which further understanding for species diversity maintenance with global change (Hassan et al., 2022).

## 5 CONCLUSIONS

Our study highlights intraspecific variation in JC effects, where *P. pinnata* have less defenses to their natively‐sourced soil, but are more defensive to the soil feedbacks from soil sourced from other populations. We cautiously interpret that the effects were driven by the differential negative impacts of soil pathogens among populations. Moreover, the strength of PSFs resulting from JC effects was driven by both the abiotic and biotic environment. Thus, global change‐related plant stressors (such as increasing drought) could potentially weaken such effects, which may lead to a potential for diversity loss at local to scales, especially in tropical ecosystems.

## AUTHOR CONTRIBUTIONS

**Libing Pan:** Conceptualization (equal); data curation (equal); investigation (equal); writing – original draft (equal). **J. Aaron Hogan:** Writing – review and editing (equal). **Xiaoyang Song:** Conceptualization (equal); data curation (equal); formal analysis (equal); funding acquisition (equal); investigation (equal); methodology (equal); supervision (equal); writing – review and editing (equal). **Wenfu Zhang:** Data curation (equal); investigation (equal); project administration (equal); writing – review and editing (equal). **Huaze Zhou:** Resources (equal). **Zhonglin Chen:** Resources (equal). **Jie Yang:** Supervision (equal); writing – review and editing (equal). **Min Cao:** Supervision (equal); writing – review and editing (equal).

## CONFLICT OF INTEREST STATEMENT

None declared.

### OPEN RESEARCH BADGES

This article has earned an Open Data badge for making publicly available the digitally‐shareable data necessary to reproduce the reported results. The data is available at [10.57760/sciencedb.17001].

## Data Availability

The data are available from the science Data Bank (https://www.scidb.cn/en) DOI: 10.57760/sciencedb.17001.
